# A Novel Differential Predict Model Based on Matrix-Assisted Laser Ionization Time-of-Flight Mass Spectrometry and Serum Ferritin for Acute Graft-versus-Host Disease

**DOI:** 10.1155/2013/563751

**Published:** 2013-10-01

**Authors:** Chun-yan Zhang, Shu-hong Wang, Wen-rong Huang, Guang-hong Guo, Zhu-hong Zhang, Wen-jun Mou, Li Yu, Ya-Ping Tian

**Affiliations:** ^1^Department of Clinical Biochemistry, Chinese PLA General Hospital, 28 Fu-Xing Road, Beijing 100853, China; ^2^Department of Immunology, Nankai University School of Medicine, Tianjin 300071, China; ^3^Department of Hematology, Chinese PLA General Hospital, 28 Fu-Xing Road, Beijing 100853, China

## Abstract

Clinical diagnosis of acute graft-versus-host disease (aGVHD) mainly depends on clinical manifestation and tissue biopsies, leading to a delayed diagnosis and treatment for aGVHD patients when the early symptom is insignificant. Our objective was to investigate the possibility of prewarning the risk of aGVHD before and after allogeneic hematopoietic stem cell transplantation (allo-HSCT) by serum protein profiling combined with serum ferritin. The difference in polypeptide expression before and after transplantation had been compared by using CLINPROT technology, and serum ferritin levels have been analyzed simultaneously. Through combining serum ferritin and MS spectral data, the diagnosis sensitivity and specificity of our model for prewarning severe aGVHD (III~IV°aGVHD) before transplant all increased to 90.0%, while after transplant, the sensitivity and specificity are 78.3% and 86.4%. Our joint prewarning model could predict the risk of aGVHD, especially severe aGVHD before and after transplant, which also provides a reliable method to the continuous monitoring of the condition of patients.

## 1. Introduction 

Allo-HSCT, as a great progress in the medical field for nearly half a century, is the most effective treatment for hematological malignancies. However, aGVHD following allo-HSCT is a major complication of restricting allo-HSCT application and curative effect, with an incidence rate of 35% to 64%. Therefore, early diagnosis and correct treatment of aGVHD have been an important topic in the field of transplantation immunology. At present, the clinical diagnosis of aGVHD mainly is based on pathological, biochemical, and histological symptoms. aGVHD usually occurs in the early stage after allo-HSCT, when most of the patients have a poor constitution and hard to tolerate tissue biopsies. Meanwhile, early performance of aGVHD is not typical, may be only skin itching, rash, mild nausea, or diarrhea, which is difficult to be diagnosed as aGVHD. But once the clinical performance is significant, the immune response has been so strong that it is dangerous and hard to be controlled even by strengthening the immune inhibitor. Therefore, noninvasive early warning and early diagnosis of aGVHD are particularly important to reduce mortality. Cytokines have been reported to be involved in the immune effect of aGVHD [1]. However, they have not yet found a suitable noninvasive blood index or portfolio to help early warning and diagnosis of aGVHD.

The use of immunosuppressive agents after allo-HSCT for treatment and prevention of aGVHD is an empiric therapy. Immune inhibitor combination based on cyclosporine A is used to prevent severe aGVHD [2]. It has been proven clinically that most of the aGVHD occurred in the prophylactic immunosuppression reducing or stop process. Up to now, there are few monitoring indicators, which can provide the basis for clinical medication [3]. Therefore, effective treatment monitoring index of aGVHD is important for early warning aGVHD and monitoring condition of patients after transplant [4].

The emergence and development of serum proteomics make the detection of protein biomarkers become of high throughput and high efficiency. Presently, researchers aboard have begun to investigate the mechanism of aGVHD and the early diagnosis of aGVHD using protein chips and show a good prospect [5, 6], but there are few reports at home. Recently, MALDI-TOF-MS technology has been widely used for the detection and identification of peptides, depending on its high sensitivity and efficiency.

Ferritin, as a principal protein for iron storage, participates in the regulation of hematopoiesis and immune system and is associated with many diseases. Almost all conditions of iron deficiency can cause ferritin reduction. The increase of serum ferritin (SF) level may be caused by blood overtransfusion, inflammation, malignant lesions, or liver diseases. A recent study showed that iron overload increases the risk of hepatic dysfunction and infections after transplantation [7].

Here, we enriched serum polypeptide from the patients with allo-HSCT and compared the difference in polypeptide expression before and after transplantation using CLINPROT technology. We compared the difference in polypeptides expression between aGVHD and non-GVHD patients and held a statistical analysis on serum ferritin levels simultaneously. By combining MS spectrum and serum ferritin, we constructed a novel prewarning model for aGVHD while avoiding invasive tissue biopsies and evaluating therapeutic effect.

## 2. Patients and Methods

### 2.1. Patients

Patients in the study were pathologically diagnosed as acute myelocytic leukemia (AML), chronic myeloid leukemia (CML), or myelodysplastic syndrome (MDS) and accepted allo-HSCT at Chinese PLA General Hospital from March 2012 till March 2013. All the patients accepted pretreatment with total body irradiation (TBI)/cyclophosphamide (Cy) scheme or modified busulfan (Bu)/Cy scheme 8~10 days before stem cell transplant. Then, the patients were treated with classic scheme to prevent aGVHD (cyclosporine A+ short-course methotrexate (MTX) + mycophenolate (MMF)). The diagnosis of aGVHD was determined by the clinical and pathologic evaluation of the patient, and aGVHD was graded according to previously published standard criteria (Table 1).

We used a total of 24 available cases for analysis with complete clinical data, among which there are 18 males, 6 females with age from 14 to 53 years, average 33.5.

**Figure 1:**
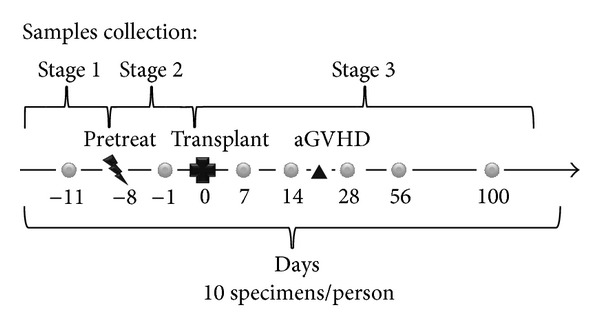
Blood specimens collection at the sequential time points.

Peripheral blood specimens from these patients were collected sequentially before and after allo-HSCT (Figure 1). For the patients of aGVHD, we collected peripheral blood specimens 1 day before treatment and 14, 28 days after treatment. We used a total of 245 specimens in mass spectrometry analysis.

### 2.2. Serum Ferritin Assay

The fasting blood was taken from cubital vein from every patient at the time point shown in Figure 1. The serum was segregated, and the biochemical parameter serum ferritin (SF) was assayed with commercially available kits (Roche Diagnostic, Penzberg, Germany) using Roche ELC2010 electrochemical luminescence instrument (Roche Diagnostic, Penzberg, Germany).

### 2.3. Blood Specimens

The blood specimens were collected from each participant within 24 hours. Fast sera were isolated by centrifugation at 4000 rpm for 7 min at 25°C and were frozen in aliquots of 150 *μ*L at −80°C immediately for use. 

### 2.4. MS Analysis

Serum peptides were enriched by weak cation exchange magnetic bead based kits (ClinProt Kits, Bruker Daltonics Inc., Fremont, CA) following the manufacturer's protocol, and spectra was acquired by matrix-assisted laser desorption/ionization time-of-flight mass spectrometry (MALDI-TOF-MS, Autoflex, Bruker Daltonik). Parameters were as follows: source 1,120 kV; ion source 2,186 kV; lens 7.6 kV; positive ion mode; 400 laser shots each sample.

### 2.5. Data Processing

ClinProTools bioinformatics software (ver. 2.0; Bruker Daltonics) was used for statistical analysis and the recognition of peptide patterns. The MS spectra for peaks of 1,000–10,000 *m*/*z* (with a signal-to-noise ratio >5) were selected and calculated by *t-*test *P* value/analysis using SPSS software (ver.19.0). The proteins/peptides with a *P* value < 0.05 were confirmed to be significantly different.

## 3. Results

### 3.1. The Distribution of aGVHD Group and Non-GVHD Group

**Figure 2:**
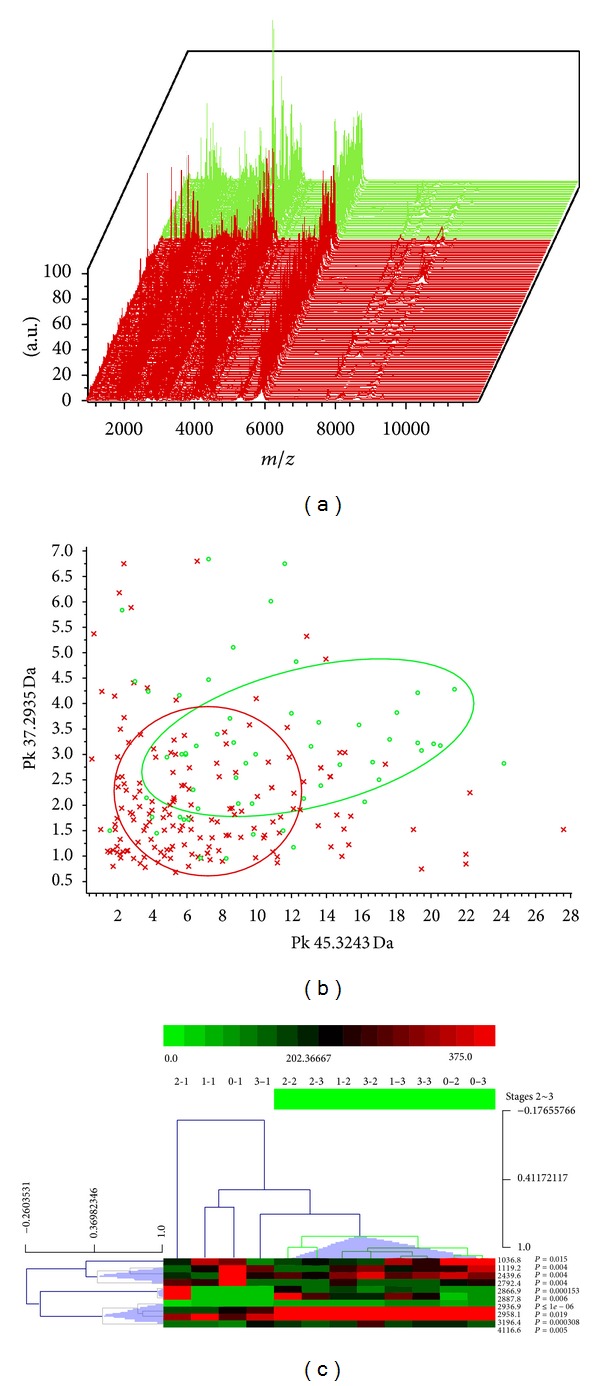
The distribution of aGVHD and non-GVHD patients. (a) Three-dimensional *m*/*z* ratio-intensity maps of aGVHD (red) and non-GVHD (green) specimens; (b) classification effect of the first two peaks (2935.4 Da and 3245.6 Da) from list of *P* values (red spots, aGVHD; green spots, non-GVHD); (c) hierarchical cluster analysis of 3-stage specimens with a set of 10 peaks.

The characteristics of the aGVHD and non-GVHD patients are shown in Table 2. The aGVHD patients included 12 males and 6 females, whose median age was 33 years (range: 15–53 years). All 245 serum specimens were analyzed by MADLI-TOF-MS (mass 1,000~10,000 Da; signal-to-noise ratio >5) (Figure 2(a)) and distributed between non-GVHD and aGVHD by peak 2935.4 Da and 3245.6 Da (Figure 2(b)). All these serum specimens were collected from stage 1 to stage 3 (Figure 1), and a set of 10 peaks that discriminate the 3 stages were used in the hierarchical cluster analysis. As shown in Figure 2(c), serum peptides/proteins in stage 1 are inconsistent with the latter two stages, suggesting that the grouping should be peptides should be grouped and analyzed base on the treatment stages. 

**Figure 3:**
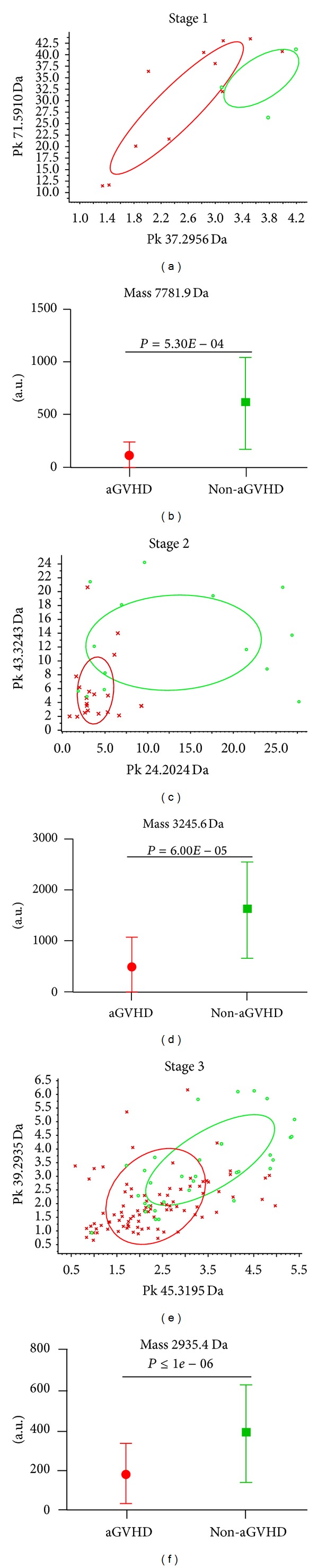
The distribution of aGVHD and non-GVHD patients in stage 1, stage 2, and stage 3 (red, aGVHD; green, non-GVHD). (a) classification effect of the first two peaks (5910 Da; 2957.7 Da) from list of *P* values in stage 1; (b) comparison of the expression of peak 7781.9 Da between aGVHD and non-GVHD patients in stage 1, *P* = 5.30*E* − 04; (c) classification effect of the first two peaks (3245.6 Da; 2026 Da) in stage 2; (d) comparison of the expression of peak 3245.6 Da in stage 2, *P* = 6.00*E* − 05; (e) classification effect of the first two peaks (2935.4 Da; 3195.6 Da) in stage 3; (f) comparison of the expression of peak 2935.4 Da in stage 3, *P* < 1*e* − 06.

Figure 3 compares the different serum proteins/peptides between aGVHD and non-GVHD in stage 1 (before pretreatment), stage 2 (before transplant), and stage 3 (after transplant), respectively. *t*- and Mann-Whitney *U* tests were performed to determine significant differences between the aGVHD and non-GVHD groups (Table 3), and we made rough estimate of the patients into aGVHD and non-GVHD group using mass spectrometry results (Figures 3(a), 3(c), and 3(e)). We showed different expression of peptides mass 7781.9 Da (*P* = 5.30*E* − 04) (Figure 3(b)), 3245.6 Da (*P* = 6.00*E* − 05) (Figure 3(d)), and 2935.4 Da (*P* < 1*e* − 06) (Figure 3(f)) between aGVHD and non-GVHD patients in different stages. 

### 3.2. Modeling for aGVHD and Non-GVHD with Mass Spectra Data

**Figure 4:**
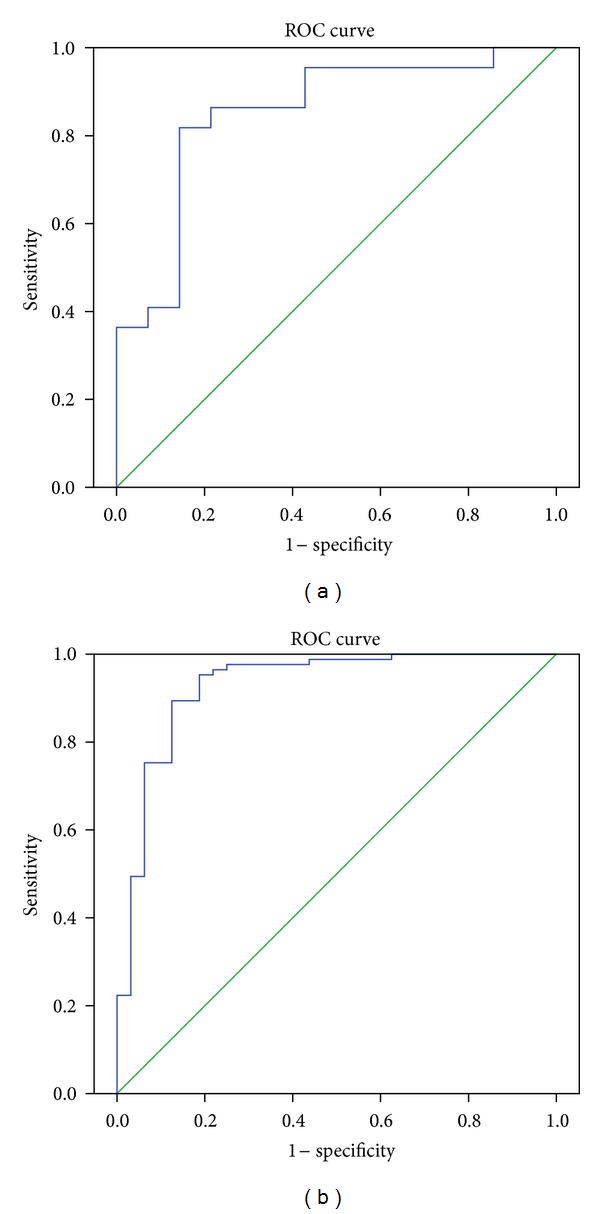
Respective ROC curve of the predicted probability of aGVHD in stage 2 (a) and stage 3 (b) with MS data from logistic regression. The state variable is aGVHD.

For the purposes of prewarning aGVHD, we constructed models in stage 2 and stage 3 using binary logistic regression analyses. Previously, we analyzed the correlation of the peaks selected by ClinProTools software and substituted each peptide into a binary logistic regression (method “Enter”) for their contribution to our model (data not shown). The peptides with a significant contribution to the model (*P* < 0.01) and relatively independent to each other (*r* < 0.7, *P* < 0.01) were substituted into the multivariate logistic regression (Table 4). As shown in Figure 4(a), only one peptide 3245.6 Da enters the model for predicting aGVHD and non-GVHD in stage 2. The area under the curve of ROC curve (AUROC) is 0.851, and accuracy is 77.8%, with a sensitivity of 81.8% and a specificity of 85.7%. Meanwhile, there are 3 peptides (1264.6 Da, 2740.8 Da, and 2935.4 Da) participating in the predict model for aGVHD and non-GVHD during stage 3. AUROC is up to 0.929 (Figure 4(b)), accuracy is 90.6%, while the sensitivity and specificity are 89.4% and 87.5%. These data demonstrate the capability of mass spectra data to predict aGVHD during a certain period. The classification equation for predict aGVHD is as follows:

Stage  2: *P* = [1+*e* ^(−0.002×mass3245.6+2.346)^  ]^−1^,
stage  3: *P* = [1 + (*e* ^(−0.021×mass1264.6+0.0979×mass2740.8)^ × *e* ^(−0.012×mass2935.4−1.225)^)]^−1^.

### 3.3. Modeling for Severe aGVHD

**Figure 5:**
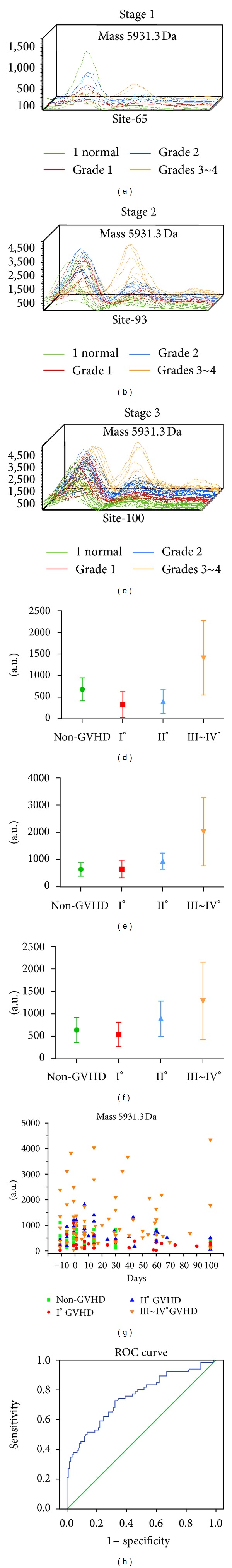
The expression of peak 5931.3 Da in different grades of aGVHD. (green: non-GVHD, red: I°aGVHD, blue: II°aGVHD, and yellow: III~IV°aGVHD). ((a)~(c)) Three-dimensional *m*/*z* ratio-intensity maps of peak 5931.3 Da in stage 1(a), stage 2(b), and stage 3(c); ((d)~(f)) bar chart of peak 5931.3 Da intensity in the four different groups, showing an increasing trend in peak 5931.3 Da in severe aGVHD patients in all 3 stages (*P* < 0.01); (g) the expression of peak 5931.3 Da in all the patients through the whole process of allo-HSCT; (h) ROC curve of the predicted probability of severe aGVHD with peak 5931.3 Da from logistic regression.

Patients with severe aGVHD not responding to treatment with steroids have a poor prognosis [8]. Although the incidence of III~IV°aGVHD is low, clinical data has shown that it almost causes an irrevocable threat to the lives of the patients. Therefore, prewarning severe aGVHD is related to minimizing the incidence and improving the prospects of survival directly [9]. We further refined the groups into I°aGVHD, II°aGVHD, III~IV°aGVHD, and non-GVHD depending on clinical consensus criteria for staging of aGVHD (Table 1) and contrasted the serum peptides/proteins difference of the four groups. In the peaks which showed significant differences analyzed by ClinProTools software (Table 5), the peak 5931.3 Da shows favourable value on prewarning III~IV°aGVHD throughout the whole process of treatment. Figures 5(a)–5(c) show the intuitive peaks of the four groups, and Figures 5(d)–5(f) illustrate the intensities of 5931.3 Da peak separately in 3 stages. Peak 5931.3 Da shows a marked overexpression in III~IV°aGVHD group, compared to non-GVHD group, I°aGVHD group, and II°aGVHD group (Figure 5(g)). When the peptide mass 5931.3 Da was used to distinguish III~IV°aGVHD patients from non-GVHD and I~II°aGVHD patients, AUROC was 0.754 (Figure 5(h)). 

Most of the severe aGVHD occurred in the prophylactic immunosuppression reducing or stop process, which caused by empirical drug stop and the lack of effective indicators for early warning aGVHD. Therefore, our models should double as an effective treatment monitoring index of severe aGVHD. We grouped non-GVHD and I~II°aGVHD to distinguish severe aGVHD from them. The correlation of the peaks and their contribution to predict models were analyzed as before, and peptides with a significant effect were substituted into the multivariate logistic regression (Table 6).

**Figure 6:**
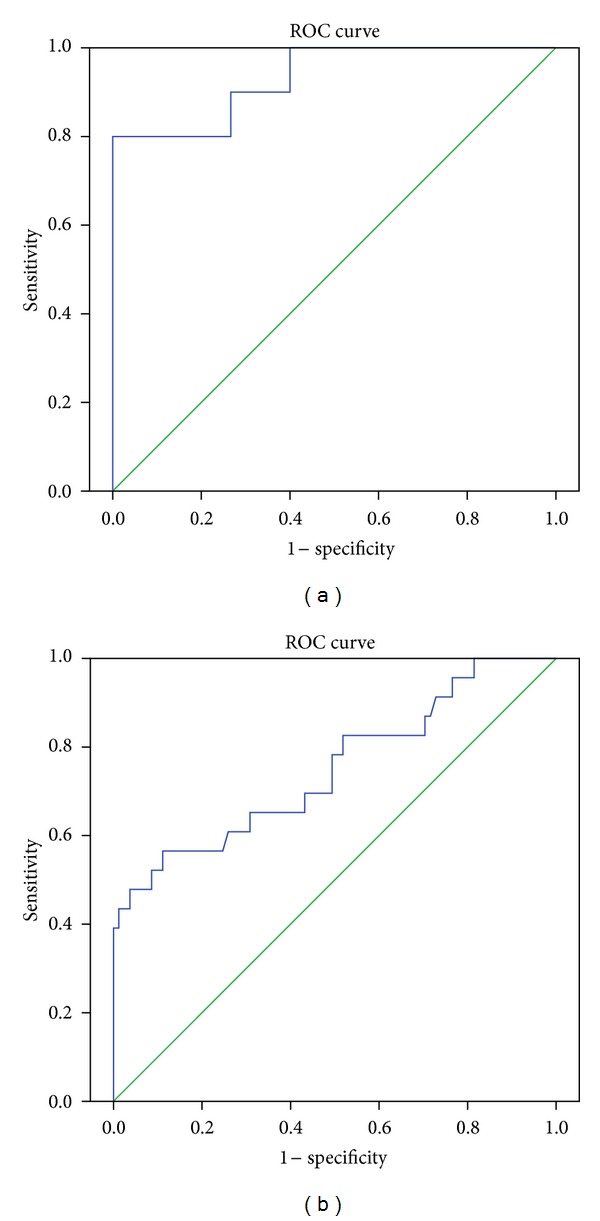
Respective ROC curve of the predicted probability of severe aGVHD in stage 2 (a) and stage 3 (b) with MS data from logistic regression. The state variable is severe aGVHD.

During the stage from pretreatment to transplant, the model for III~IV°aGVHD and non-GVHD, I~II°aGVHD, and AUROC of the unite peak, which combined with peaks 2809.6 Da, and 3976.9 Da is up to 0.933. The accuracy is 95.0% with a sensitivity of 80.0% and a specificity of 100.0% (Figure 6(a)). Meanwhile, during the stage from transplant to aGVHD outbreak, AUROC of the unite model (peaks 2815.4 Da, 3995.6 Da, 4303.2 Da, and 5931.3 Da) is 0.750, accuracy is 85.6%, and the sensitivity and specificity of it are 56.5% and 88.9%, separately (Figure 6(b)). The two models can predict the risk of progression to severe aGVHD in stage 2 and stage 3, respectively.

The peptides in our models such as mass 1264.6 Da and 3245.6 Da have been identified, and they belong to fibrinogen alpha chain precursor [10, 11]. The others are still unknown. However, it is essential to verify them, especially 5931.3 Da, which has a high contribution to aGVHD.

### 3.4. Value of Serum Ferritin to Predict aGVHD

**Figure 7:**
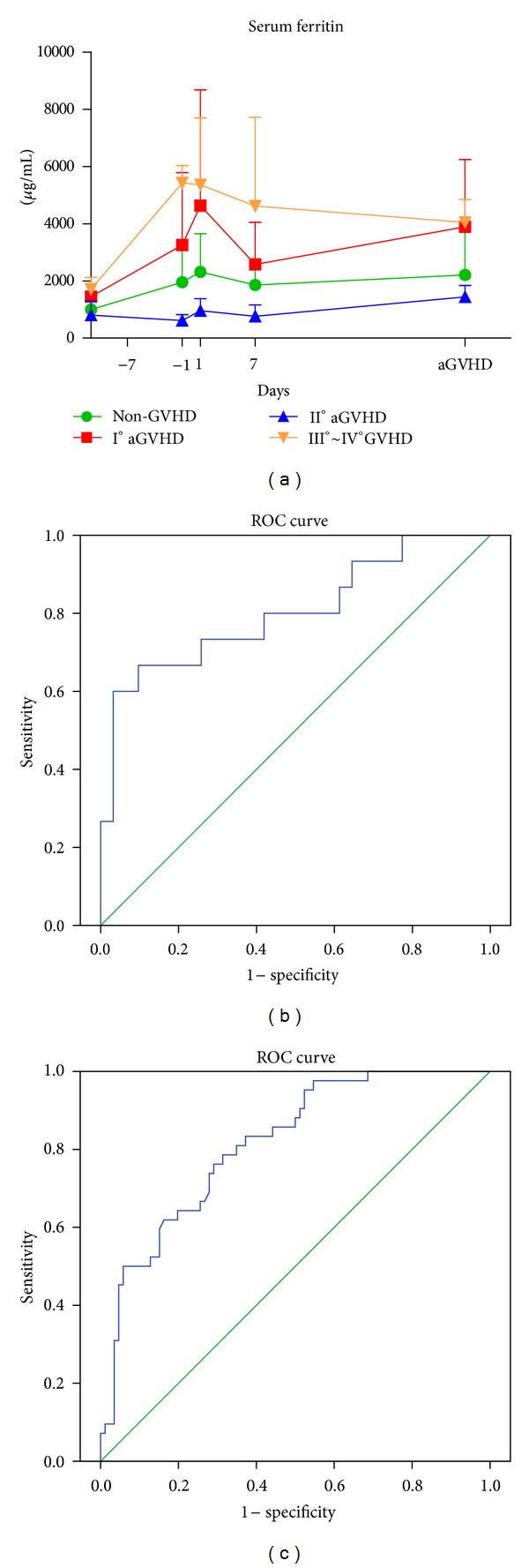
The effect of serum ferritin level on predicting severe aGVHD. (a) The level of serum ferritin in non-GVHD (green), I°aGVHD (red), II°aGVHD (blue), and III~IV°aGVHD (yellow); ((b)~(c)) respective ROC curve of the predicted probability of severe aGVHD with single serum ferritin in stage 2 (b) and stage 3 (c) from logistic regression. The state variable is severe aGVHD.

Due to the unavoidable noise of the mass spectra coming from instrument and other external interference, serum ferritin is involved into our models to induce noise and improve the sensitivity. Iron overload is frequently observed in patients with hepatopathy, cancer, and hematologic diseases, and serum ferritin is the most sensitive indicator for iron metabolism. Previous studies have shown that, during allo-HSCT, elevated pretransplant serum ferritin level is associated with a higher incidence of treatment-related complications [7, 12–14]. However, the specificity and sensitivity of sole serum ferritin to aGVHD are very limited due to individual diversity. The normal values of serum ferritin in biochemical tests are male (20~60 years old) 30~400 ng/mL; female (17~60 years old) 13–150 ng/mL. As shown in Figure 7(a), statistics suggests that I°aGVHD and III~IV°aGVHD groups, especially the latter, have an elevated serum ferritin level before and after transplant. Therefore, we used serum ferritin to predict severe aGVHD instead of all grades of aGVHD. During stage 2, AUROC of single SF is 0.802 (Figure 7(b)), with a sensitivity of 66.7% and a specificity of 90.3%; in stage 3, AUROC of single SF is 0.814 (Figure 7(c)), with a sensitivity of 78.3% and a specificity of 77.8%.

### 3.5. The Combination Model for Prewarning Severe aGVHD

**Figure 8:**
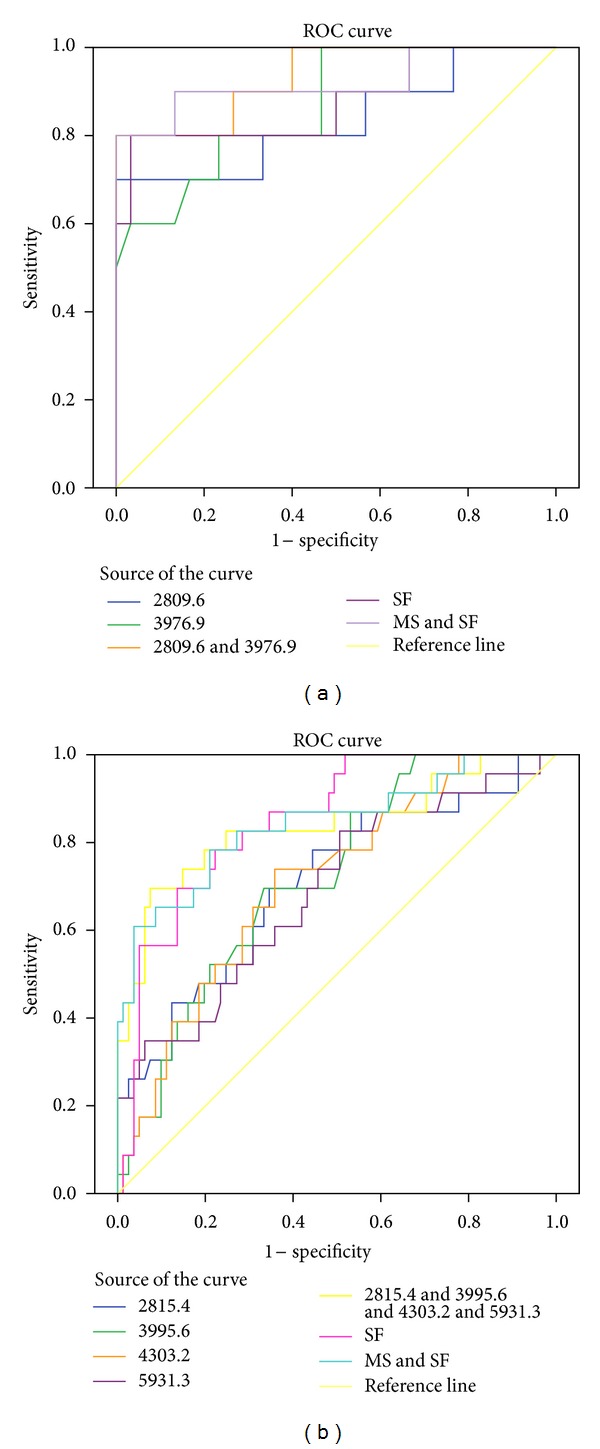
Respective ROC curve of the predicted probability of severe aGVHD with “MS+SF” combination in stage 2 (a) and stage 3 (b) from logistic regression. The state variable is severe aGVHD.

To improve the prewarning value of mass spectrometry and serum ferritin to aGVHD, we conjointly analyzed the two noninvasive indicators (Table 7). Figures 8(a) and 8(b) illuminate that the sensitivity and specificity are obviously elevated using added method. During the period from pretreatment to transplant, AUROC of combination is 0.920 and accuracy is 90%, with a sensitivity of 90.0% and a specificity of 90.0% (Figure 8(a)). After transplant, the conjoint analysis of mass spectrometry and serum ferritin also has an elevated early-warning value. AUROC of it is 0.855 and accuracy is 85.6%, the sensitivity and specificity of it are 78.3% and 86.4% (Figure 8(b)). This further develops the potential of our data and supports our methods that are robust for raw MS data preprocessing.

The classification equation combined with mass spectrometry and serum ferritin for predicting severe aGVHD is as follows:

stage  2: *P* = [1 + (*e* ^(0.014×mass2809.6+0.009×mass3976.9)^ ×  *e* ^(+0.001×SF−6.666)^)]^−1^  ,
stage  3: *P* =  [1 + (*e* ^(0.004×mass2815.4+0.004×mass3995.6)^ × *e* ^(+0.002×mass4303.2+0.001×mass5931.3+0.001×SF−4.970)^)]^−1^.

## 4. Discussion

Although the pathophysiology of acute GVHD is complex, accumulating evidence suggests that most of the effectors participating in aGVHD immune responses are serum peptides/proteins [4]. Most previous investigations focused on cytokine in aGVHD and made it as biomarker [6, 15–17], which is reliable, stable, but lagging. In another words, cytokine is not effective enough for prewarning aGVHD. Therefore, we collected blood samples from the patients at sequential points from the very beginning of the treatment to aGVHD occurrence for our research.

Currently, MALDI-TOF-MS has been one of the sensitive and effective approaches for identifying potential biomarkers of health and disease [18]. Here, we enriched the serum low abundance peptides/proteins by WCX magnetic beads and got serum protein profiling by MALDI-TOF-MS, which offers wide selection, and at the same time it is a noninvasive detection avoiding tissue biopsies. 

In this study, we analyzed the difference between aGVHD and non-GVHD group before and after transplantation. During the stage from pretreatment to transplant, our MS spectral data shows prewarning potential to aGVHD. Before transplant, the sensitivity and specificity of our model are 81.8% and 85.7%, which serve as an early warning of aGVHD, giving therapists plenty of time to make preventive measures. After transplant, the accuracy of our model is 90.6%, while the sensitivity and specificity are 89.4% and 87.5%. 

Due to individual diversity and instrument noise, raw MALDI-TOF MS spectra need to be analyzed based on clinical features. Therefore, we constructed the models combined with serum ferritin. Iron overload increases the risk of infections, venoocclusive disease and hepatic dysfunction in posttransplant period [12], which is also proved by our statistical data. Our statistics reports that SF concentrations of our patients all exceed the upper line. Moreover, SF levels of the patients with aGVHD, especially severe aGVHD (III~IV°aGVHD) are much higher than those of non-GVHD group, whether before transplant or after transplant. Clinical statistics have reported that severe intestinal aGVHD was often difficult to reverse and sometime rapidly lead to death. Through combining serum ferritin and MS spectral data, the sensitivity and specificity of our model for prewarning severe aGVHD before transplant increased to 90.0%. The warning during this stage will provide sufficient time for therapists to observe the patient closely; to start, stop, or change the dosage of any medicine; to delay or stop transplant; and to enhance preparedness and aGVHD protection.

After transplant, there is also joint scheme that suggests a risk for severe aGVHD, the sensitivity and specificity of which are 78.3% and 86.4%. In clinically work, the dosage of immunosuppressive drugs prone to be insufficient or excessive, since of that treatment response and monitoring indicators are insufficient at present. Short dosage would cause disease, like aGVHD, while excess drugs would lead to a tumor recurrence. [3]. Our model can realize continuous monitoring to the condition of patients and prewarning the risk for severe aGVHD, which threatens the lives of the patients.

An interesting phenomenon in our models was that the peak 3245.6 Da, which is a fragment of fibrinogen alpha chain precursor, was more highly expressed in non-GVHD than in aGVHD patients before transplant. This suggested the potential of 3245.6 Da as a biomarker for aGVHD and the possible relationship between fibrinolysis and aGVHD, which would be explored in our further work. The peak 5931.3 Da in the original MALDI-TOF profiles showed its trend to severe aGVHD through the entire course of allo-HSCT. Further identification is needed for this biomarker for severe aGVHD, and intensive study remains to be continued.

Taken together, all of the evidences reveal a novel prewarning model for aGVHD while avoiding invasive tissue biopsies and improving long-term effect of transplantation. Furthermore, more studies are needed to verify the special serum peptides expression and the roles they play.

## 5. Conclusions

Our models can predict aGVHD and non-GVHD based on MS spectral data while avoiding invasive tissue biopsies. The sensitivities of the models are 81.8% and 89.4% before and after transplant. Moreover, through combining serum ferritin and MS spectral data, the sensitivity and specificity of our model for prewarning severe aGVHD (III~IV°aGVHD) before transplant all increased to 90.0%, while after transplant the sensitivity and specificity are 78.3% and 86.4%. 

## Figures and Tables

**Table 1:** Consensus criteria for staging of aGVHD.

Grade	Skin	Liver	Gut
(I) Mild	Maculopapular skin area 25~50% of body	Normal	Diarrhea volume 500–1000 mL/day; nausea and emesis
(II) Moderate	Maculopapular skin area 50~100% of body	Serum bilirubin < 51 *μ*mol/L	Diarrhea volume 1000~1500 mL/day; nausea and emesis
(III) Severe	Maculopapular skin area 50~100% of body	Serum bilirubin 51~256 *μ*mol/L	Diarrhea volume > 1500 mL/day; nausea and emesis
(IV) Life threatening	Generalized exfoliative dermatitis or ulcerative dermatitis, simplex	Serum bilirubin > 256 *μ*mol/L	Severe abdominal pain, with bloody diarrhea or intestinal obstruction

**Table 2:** Demographic and clinical characteristics of patients with and without acute GVHD.

Case	Age	Recipient gender	Doner gender	Underlying disease	Donor type	Pretreatment	Clinically affected aGVHD organ	aGVHD grade
1	48	M	F	AML	Related	BU + CY	Skin, mouth, gastrointestinal tract	IV
2	36	M	F	AML	Related	BU + CY		0
3	25	M	M	AML	Related	BU + CY		0
4	47	M	M	AML	Related	BU + CY	Skin, mouth	0
5	53	F	F	CML	Related	Flud + BU	Gastrointestinal tract	III
6	14	M	M	ALL	Related	TBI + CY + ATG		0
7	31	F	F	AML	Unrelated	BU + CY	Skin	I
8	15	M	M	AML	Related	BU + CY + ATG	Skin, liver	II
9	40	M	M	MDS	Related	BU + CY + ATG	Skin, mouth, gastrointestinal tract	IV
10	22	M	M	ALL	Related	TBI + CY + ATG	Skin, mouth, liver, gastrointestinal tract	III
11	22	M	M	MDS	Unrelated	BU + CY + ATG	Skin, mouth, gastrointestinal tract	IV
12	44	F	F	AML	Unrelated	BU + CY + ATG	Gastrointestinal tract	II
13	28	M	M	ALL	Unrelated	TBI + CY + ATG		0
14	28	M	M	ALL	Related	TBI + CY		0
16	47	F	F	CML	Related	BU + CY	Skin, liver, gastrointestinal tract	II
17	43	M	M	MDS	Related	BU + CY + ATG	Gastrointestinal tract	II
18	44	M	F	AML	Related	BU + CY	Liver, gastrointestinal tract	III
19	49	F	F	ALL	Unrelated	BU + CY + ATG	Gastrointestinal tract	II
22	15	M	M	ALL	Unrelated	BU + CY + ATG	Skin	I
30	23	M	M	ALL	Related	Flud + BU	Gastrointestinal tract	II
33	16	M	F	MDS	Related	BU + CY + ATG	Gastrointestinal tract	I
36	43	M	M	AML	Unrelated	BU + CY + ATG	Skin	I
39	27	F	F	CML	Unrelated	BU + CY	Skin, mouth	I
40	45	M	M	MDS	Related	BU + CY	Skin	I

M: male; F: female; AML: acute myelogenous leukemia; CML: chronic myelogenous leukemia; MDS: myelodysplastic syndrome; ALL: acute lymophoblastic leukemia; TBI: total body irradiation; BU: busulfan; CY: cyclophosphamide; Flud: fludarabine; ATG: rabbit antithymocyte globulin.

**Table 3:** Statistical information for marker peptides of aGVHD and non-GVHD groups.

Stage	Mass	*P*TTA (*t*)	*P*-WTest	Ave. (aGVHD)	Ave. (non-GVHD)	SD (aGVHD)	SD (non-GVHD)
1	7781.9	5.30*E* − 04	0.005	127.4	727	101.5	386.2
	2887.8	0.023	0.021	316.1	187.8	109.5	73.2
	2957.7	0.027	0.04	235.4	428.7	97	219.5

2	3245.6	6.00*E* − 05	4.85*E* − 04	528.3	1614.9	520.8	893.9
	2866.5	0.000143	0.000694	134	483.6	106.1	363.5
	2046.6	0.004	0.051	86.8	212.4	43	183.4
	1781.8	0.004	0.058	139.7	415.5	82.7	410.8
	1351.9	0.005	0.012	60	148.4	29.5	135.9
	2026	0.006	0.048	548.5	1525.9	420.7	1468.8
	2995.1	0.008	0.000984	81.5	348.1	72.9	435.5
	8952.2	0.008	0.005	31.7	117.4	31.7	138.8
	1453.2	0.009	0.05	102.4	277.3	57	290.8
	4236.6	0.01	0.005	652.5	429.1	286	127.9
	1694.6	0.013	0.06	83.9	230.4	47.2	256.3
	1609.3	0.016	0.036	48.7	125.8	39.7	135.2
	8142.8	0.029	0.111	18.1	133.6	13.2	239.9
	5936.3	0.034	0.031	1183.9	660.4	858.9	257.6
	5928	0.04	0.039	1158.8	649.4	865.9	254.3
	3961	0.041	0.217	248.4	385.9	114	269.3
	2275.3	0.042	0.135	119.5	181.4	61.3	114.7
	1096.4	0.043	0.088	161	86	129.7	36
	4617.8	4.50*E* − 02	5.70*E* − 01	30.7	124.1	16.5	211.7

3	2935.4	<1*e* − 6	3.3*E* − 06	185.5	384.5	140.9	243
	3195.6	1.19*E* − 05	0.000112	145.7	254.3	101.9	156.4
	3474.1	0.000563	0.902	35.6	608.2	16.5	1497.9
	3246	0.000677	0.001	836.9	1477.1	772.7	1224.9
	3455	0.001	0.052	46.8	80.5	20.2	88.3
	3318.4	0.002	0.636	45.2	85.4	22.8	112.1
	4019.4	0.005	0.002	84.5	66.2	36.3	20.6
	3939.6	0.007	0.004	118.3	87.2	63.5	37.5
	1264.6	0.008	0.042	84.7	118.3	47.5	90.8
	2740.8	0.009	0.003	102	72	65.3	29.4
	3493.3	0.01	0.705	34.3	127.4	16	327.6
	5365.9	0.014	0.032	216.8	148.5	158.7	77.8
	2867.3	0.016	0.017	268.8	450.9	279.9	539.9
	1984.4	0.018	0.016	129.8	100.2	68.1	48.3
	4197.2	0.019	0.023	469	411.4	116.1	138.1
	2958.1	0.022	0.026	266.8	317.9	112.1	110.8
	3506.6	0.023	0.352	47.5	107.9	27.2	240.2
	2815.4	0.026	0.1	163	99.7	166.4	55.2
	3995.6	0.028	0.01	92	65.9	64.6	45.3
	2724.9	0.029	0.013	116.9	82.1	93.4	31.3
	1922.2	0.031	0.263	86.4	118.2	58.1	101.7
	3979.2	0.033	0.007	322.2	214.4	266	223.9
	3687.2	0.035	0.009	93.9	69.3	61.4	50.2
	3211.9	0.035	0.006	91.4	134.8	104.3	99.9
	1890	0.042	0.221	228.2	294.1	120.4	234.1
	1119.5	0.047	0.02	244.8	102.3	415.7	171.3
	5908	0.048	0.075	2074.5	2466.7	1043.6	886.9

Mass: *m*/*z*; *P*TTA: *P* value of *t*-test (two classes); *P*WKW: *P* value of Wilcoxon test (>two classes).

**Table 4:** Parameters in predict models for aGVHD and non-GVHD.

Variables in the equation									Classification table		
Stage	*B*	S.E.	Wald	df	Sig.	Exp (*B*)	95% C.I. for EXP (*B*)				Percentage correct
							Lower	Upper			
Stage 2											
3245.6	−.002	.001	9.105	1	.003	.998	.997	.999	Disease	Non-GVHD	64.3
										aGVHD	86.4
Constant	2.346	.729	10.341	1	.001	10.442			Overall percentage		77.8

Stage 3											
1264.6	−.021	.008	6.762	1	.009	.979	.964	.995	Disease	Non-GVHD	78.1
2740.8	.097	.023	18.145	1	.000	1.101	1.054	1.152		aGVHD	95.3
2935.4	−.012	.003	17.430	1	.000	.989	.983	.994			
Constant	−1.225	.922	1.765	1	.184	.294			Overall percentage		90.6

**Table 5:** Statistical information for marker peptides of I~IV°aGVHD and non-GVHD groups.

Stage	Mass	*P*TTA (*t*)	*P*- KWTest	Ave. (non-GVHD)	SD (non-GVHD)	Ave. (I°aGVHD)	SD (I°aGVHD)	Ave. (II°aGVHD)	SD (II°aGVHD)	Ave. (III~IV°aGVHD)	SD (III~IV°aGVHD)
1	1984.7	1.15*E* − 04	0.019	84.3	44.5	83.3	36.4	283	66.6	74	39.5
	3161.8	0.003	0.055	576.8	310.5	393.3	189.5	295.8	89	1167.7	388.4
	3183.8	0.013	0.083	70.8	23.4	80.5	66.8	53.8	31.4	231.3	121.5
	7781.9	0.03	0.036	686	439.5	98	99.4	292.8	290.3	20.7	23.1
	5931.3	0.035	0.127	680.8	266.8	327	300	397.8	277.9	1410.3	864.3

2	2809.6	5.91*E* − 05	0.008	117.7	57.4	117.9	71.1	117.3	77.6	408.9	252.4
	5931.3	1.39*E* − 04	0.003	641.6	249	644.7	313.9	938.3	298.6	2023.3	1253
	5362.2	5.94*E* − 04	0.012	163.3	83.3	127.9	67.3	178	113.2	420.8	259.4
	1119.5	8.73*E* − 04	0.02	47.8	20	102.7	63.5	614.8	619.4	113.8	147
	2866.5	0.003	0.005	385.1	271.9	158.1	73.2	107.5	101.5	129.4	120.2
	1781.5	0.007	0.086	459.4	429.4	100.1	64.6	126.5	86.1	157	90.3
	4233.8	0.009	0.015	449	127.6	568.1	245.5	488.1	231.3	800.6	301.9
	2046.6	0.011	0.214	226.4	195.6	70.1	28.6	72.5	21.3	106.8	55.3
	5910.2	0.014	0.026	2381.3	1018.4	2422.2	883	2753.1	728	1287.2	1036.9
	5341	0.015	0.033	605.8	243.3	525.8	264.2	507.1	224	254.4	207.6
	3245.6	0.017	0.008	1490	880.8	938.4	942.1	445.3	359.4	500.1	764.7
	1899.8	0.017	0.069	420.1	279.8	793.2	561.8	303.4	96.9	378.1	180
	2025.1	0.018	0.097	1593	1579.7	323.6	115.3	474.8	278.8	678.9	585.5
	1085.5	0.018	0.12	125.3	62.8	242.8	150.8	153.3	42.6	120.2	59.2
	1969.3	0.018	0.009	262.3	144.4	320.2	179.7	108.5	37.7	237.3	113.4
	1280.8	0.021	0.16	177.8	88.3	373.8	309.5	145.6	63.1	161.4	91.7
	8952.2	0.022	0.048	130.2	146.7	36.1	41.8	24.3	13.3	29.3	26.6
	1069.1	0.023	0.145	404.2	188.9	627.7	322.6	404.4	136.2	320	123.6
	1351.9	0.024	0.067	159.2	144.3	76.2	42.4	48.6	10.2	61.6	33.6
	5827.7	0.026	0.145	84	36.5	136.1	103.1	93.4	46	217.3	168.9
	1453.2	0.027	0.166	304	307.1	114	62.2	88.9	63.5	99.3	52.4
	3976.9	0.028	0.011	157.2	92.5	142	66.9	101.1	52.2	458.1	529.6
	1694.6	0.033	0.157	255	269.9	87.7	42.9	74.9	45.1	82.9	59.6
	5835.9	0.034	0.133	97.3	30.4	145.6	100.5	109.9	37.1	225.4	170.5
	1413.9	0.039	0.08	39.3	19.7	130.2	167.3	30.9	11.9	31.3	17.3
	1053.4	0.042	0.374	265.8	124.1	447.8	280.7	238.4	94.8	241.8	136.4
	3959.6	0.042	0.071	426.3	270.3	354.8	209.5	217.1	95.6	198.6	130.1
	1027.6	0.044	0.075	47.4	23.2	136.6	137.2	151.9	154.5	48.3	32.9
	1096.4	0.047	0.126	84.5	38.1	199.7	153.3	176.9	125.3	103.6	70.2

3	3245.6	2.35*E* − 05	6.13*E* − 06	1549.8	1274.7	817.5	742.2	753.3	597.7	349.7	366.2
	5931.3	7.44*E* − 05	2.31*E* − 04	647.7	275.3	545.5	271.3	897.3	393.8	1297	864.4
	2935.8	9.42*E* − 05	1.65*E* − 04	361.2	233.4	188.7	131.9	132.8	88.8	227.2	226.2
	2815.4	3.30*E* − 04	0.007	97.1	49.6	136	74.3	125.9	96.5	260.5	253.7
	3195.6	5.01*E* − 04	0.001	243.2	155.9	129.7	94.2	127.5	96.1	128.3	113
	5341.5	5.99*E* − 04	9.54*E* − 04	527.7	247.1	616.9	314	750.2	470.4	338.8	276.5
	3976.4	0.001	7.19*E* − 04	236.6	240.4	370.6	267.6	232.3	189.2	493.4	335.1
	5360.6	0.001	4.25*E* − 04	144.6	76.5	155.4	94.6	271.5	150.5	348	384.7
	3995.6	0.003	0.002	68.5	47.9	100	63.1	72.3	47.2	127.9	88.5
	4303.2	0.006	0.002	128.5	110.7	169.3	97.2	116.4	79.5	214.3	128
	1947.6	0.013	0.012	1574.5	1497.1	1608.5	1326.5	673.2	421.5	1106.9	1087.4
	2757.4	0.014	0.008	114.3	84	201.1	258	72.1	39.5	130.6	77.8
	2866.5	0.016	6.31*E* − 04	439.5	541.4	284.2	367.7	230.1	175.1	128.2	62.4
	1609.6	0.016	0.024	54.7	28.5	86.5	93.2	53	30.6	40.6	16.4
	2565.3	0.019	0.27	70.2	31.9	126.8	126.7	63.7	35.4	86.8	82.3
	2370.6	0.021	0.027	109.3	56.1	156.9	110.8	92	71.5	117.8	49.1
	8952.8	0.029	0.035	47.9	38.8	123.8	240.8	33	38.1	32	49.8
	3959.2	0.03	0.007	545.5	407.4	723.7	423	435.9	409.2	415.9	339
	5910.2	0.031	0.068	2351.5	916.7	2397.5	1167	2295.2	877.4	1582.6	1325.7
	2275.3	0.032	0.07	216	176.5	169.2	88.5	124.9	56.5	227.3	177.6
	4094.9	0.035	0.024	437.4	122.7	443.6	97.8	390.2	152.6	354.5	101.2
	3687.2	0.038	0.018	73.7	53.2	106.2	63.6	73.7	47.4	111.4	77.3
	5833.2	0.04	0.029	81	46.6	97.4	68.5	113.8	53.6	137	110.3
	1889.7	0.042	0.045	220.2	118.2	163.3	71	208.2	95	159.2	80
	4253.6	0.044	0.291	124.9	42.2	160.6	85.1	121	41	146.7	51.3
	2107.1	0.046	0.075	188.3	79.8	201.4	83.5	144.1	65.4	164.7	81.8
	5968.7	0.047	0.135	115.8	54.9	121.6	59.6	159.8	81.9	159.1	94.8
	1899.8	0.047	0.1	450.2	305.7	272.4	174.9	330	235.8	311.3	236.3
	1119	0.048	0.004	89.7	152.6	377	638.5	342	486.9	181.3	298.3

**Table 6:** Parameters in predict models for severe aGVHD.

Variables in the equation										Classification table		
		*B *	S.E.	Wald	df	Sig.	Exp (*B*)	95% C.I. for EXP (*B*)				Percentage correct
								Lower	Upper			
Stage 2	2809.6	.017	.008	4.960	1	.026	1.017	1.002	1.032	Disease Grades 3~4	Non-gvhd, Grade 1, Grade 2	100.0
	3976.9	.015	.009	2.754	1	.097	1.015	.997	1.032		Grades 3~4	80.0
	Constant	−7.305	2.749	7.064	1	.008	.001			Overall percentage		95.0

Stage 3	2815.4	.005	.003	2.902	1	.088	1.005	.999	1.010	Disease Grades 3~4	Non-gvhd, Grade 1, Grade 2	98.8
	3995.6	.004	.007	.328	1	.567	1.004	.990	1.019		Grades 3~4	39.1
	4303.2	.002	.004	.175	1	.676	1.002	.993	1.011			
	5931.3	.001	.001	5.507	1	.019	1.001	1.000	1.003			
	Constant	−3.991	.791	25.470	1	.000	.018			Overall percentage		85.6

**Table 7:** Parameters in combined models for severe aGVHD.

Variables in the equation										Classification table		
		*B *	S.E.	Wald	df	Sig.	Exp (*B*)	95% C.I. for EXP(*B*)				Percentage correct
								Lower	Upper			
Stage 2	2809.6	.014	.008	2.960	1	.085	1.014	.998	1.030	Disease Grades 3~4	Non-gvhd, Grade 1, Grade 2	96.7
	3976.9	.009	.008	1.206	1	.272	1.009	.993	1.025		1.00	70.0
	SF	.001	.001	1.141	1	.285	1.001	1.000	1.002			
	Constant	−6.666	2.417	7.607	1	.006	.001			Overall percentage		90.0

Stage 3	2815.4	.004	.003	1.483	1	.223	1.004	.998	1.010	Disease Grades 3~4	Non-gvhd, Grade 1~2	96.3
	3995.6	.004	.008	.232	1	.630	1.004	.988	1.021		Grades 3~4	47.8
	4303.2	.002	.005	.180	1	.671	1.002	.992	1.012			
	5931.3	.001	.001	2.536	1	.111	1.001	1.000	1.002			
	SF	.001	.000	8.998	1	.003	1.001	1.000	1.001			
	Constant	−4.970	.958	26.914	1	.000	.007			Overall percentage		85.6

## References

[1] Bacigalupo A (2011). Acute graft-versus-host disease. *Immunotherapy*.

[2] Perez L, Anasetti C, Pidala J (2011). Have we improved in preventing and treating acute graft-versus-host disease?. *Current Opinion in Hematology*.

[3] Korngold R, Antin JH (2009). Biology and management of acute graft-versus-host disease. *Cancer Treatment and Research*.

[4] Toubai T, Tanaka J, Paczesny S, Shono Y, Reddy P, Imamura M (2012). Role of cytokines in the pathophysiology of acute graft-versus-host disease (GVHD) are serum/plasma cytokines potential biomarkers for diagnosis of acute GVHD following allogeneic hematopoietic cell transplantation (Allo-HCT)?. *Current Stem Cell Research and Therapy*.

[5] Socié G (2009). Graft-versus-host disease: proteomics comes of age. *Blood*.

[6] Paczesny S, Krijanovski OI, Braun TM (2009). A biomarker panel for acute graft-versus-host disease. *Blood*.

[7] Sakamoto S, Kawabata H, Kanda J (2013). Differing impacts of pretransplant serum ferritin and C-reactive protein levels on the incidence of chronic graft-versus-host disease after allogeneic hematopoietic stem cell transplantation. *International Journal of Hematology*.

[8] Kobbe G, Schneider P, Rohr U (2001). Treatment of severe steroid refractory acute graft-versus-host disease with infliximab, a chimeric human/mouse antiTNF*α* antibody. *Bone Marrow Transplantation*.

[9] Rezvani AR, Storer BE, Storb RF (2011). Decreased serum albumin as a biomarker for severe acute graft-versus-host disease after reduced-intensity allogeneic hematopoietic cell transplantation. *Biology of Blood and Marrow Transplantation*.

[10] Tao YL, Li Y, Gao J (2012). Identifying FGA peptides as nasopharyngeal carcinoma-associated biomarkers by magnetic beads. *Journal of Cellular Biochemistry*.

[11] Kaneshiro N, Xiang Y, Nagai K (2009). Comprehensive analysis of short peptides in sera from patients with IgA nephropathy. *Rapid Communications in Mass Spectrometry*.

[12] Sivgin S, Baldane S, Kaynar L (2012). Pretransplant serum ferritin level may be a predictive marker for outcomes in patients having undergone allogeneic hematopoietic stem cell transplantation. *Neoplasma*.

[13] Majhail NS, Lazarus HM, Burns LJ (2008). Iron overload in hematopoietic cell transplantation. *Bone Marrow Transplantation*.

[14] Armand P, Kim HT, Cutler CS (2007). Prognostic impact of elevated pretransplantation serum ferritin in patients undergoing myeloablative stem cell transplantation. *Blood*.

[15] Resende RG, Correia-Silva JD, Silva TA (2012). Saliva and blood interferon gamma levels and IFNG genotypes in acute graft-versus-host disease. *Oral Diseases*.

[16] Tawara I, Koyama M, Liu C (2011). Interleukin-6 modulates graft-versus-host responses after experimental allogeneic bone marrow transplantation. *Clinical Cancer Research*.

[17] Bouazzaoui A, Spacenko E, Mueller G (2009). Chemokine and chemokine receptor expression analysis in target organs of acute graft-versus-host disease. *Genes and Immunity*.

[18] Aldred S, Grant MM, Griffiths HR (2004). The use of proteomics for the assessment of clinical samples in research. *Clinical Biochemistry*.

